# Supplementary material for: Secret Recipe Revealed: Chemical Evaluation of Raw Colouring Mixtures from Early 19th Century Moravia
Source: Molecules. 2022 Aug 15;27(16):5205. doi: 10.3390/molecules27165205 (PMC9415618; doi:10.3390/molecules27165205)
Supplement: Supplementary file 1 [file molecules-27-05205-s001.zip › molecules-1836202-supplementary.pdf]

Secret Recipe Revealed: Chemical Evaluation of Raw Colouring Mixtures from Early 19th Century Moravia

Klára Jagošová, Martin Moník, Jaroslav Kapusta, Pavel Fojtík, Jana Nádvorníková, Radka Pechancová, Ondřej Kurka, Tereza Závodná, Petr Bednář, Lukáš Richtera, Lukáš Kučera

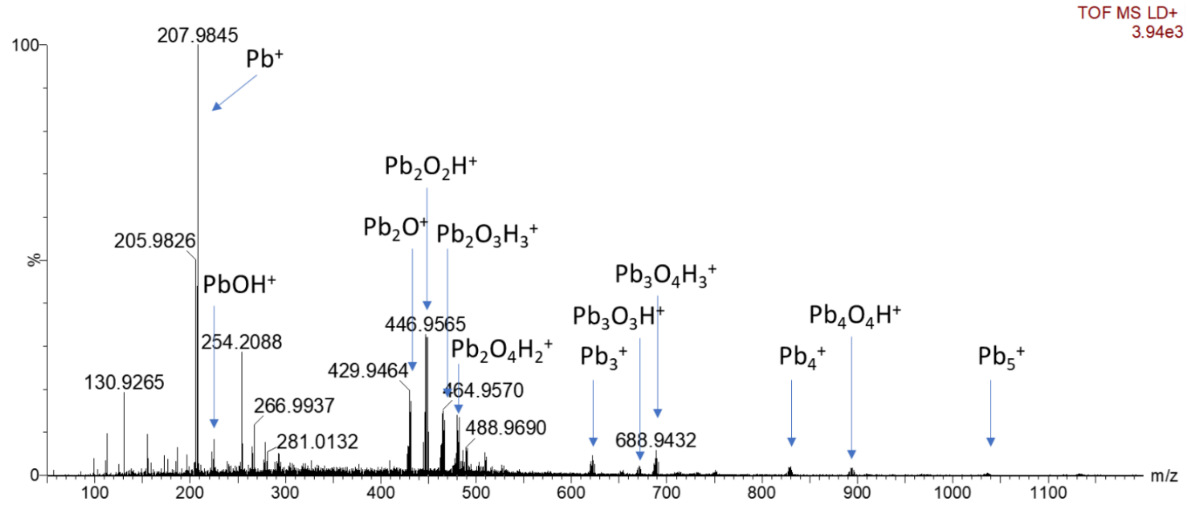

Figure S1: *LDI-MS spectra of the pink powder in positive ion mode.*

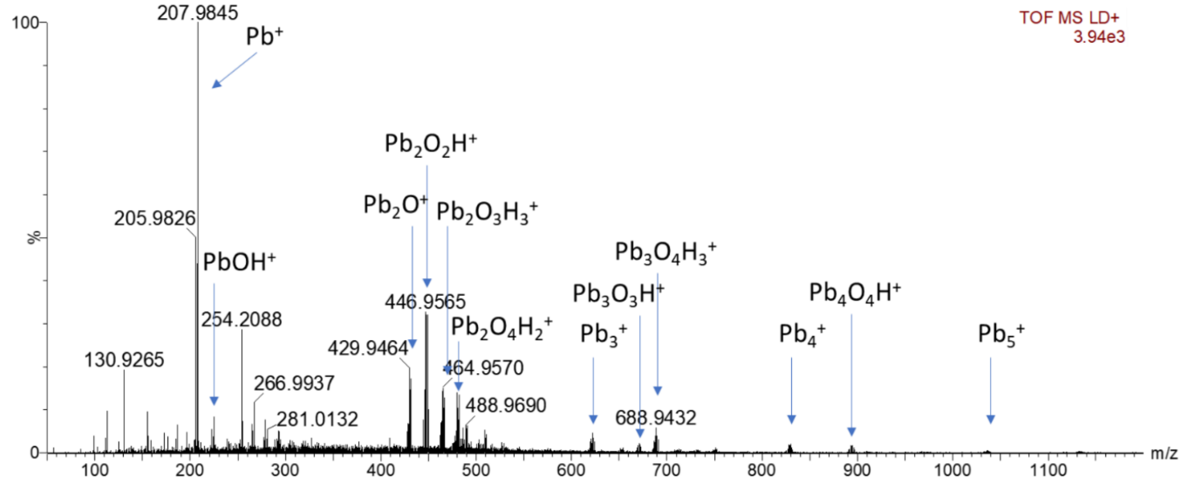

Figure S2: *LDI-MS spectra of the blue powder in positive ion mode.*

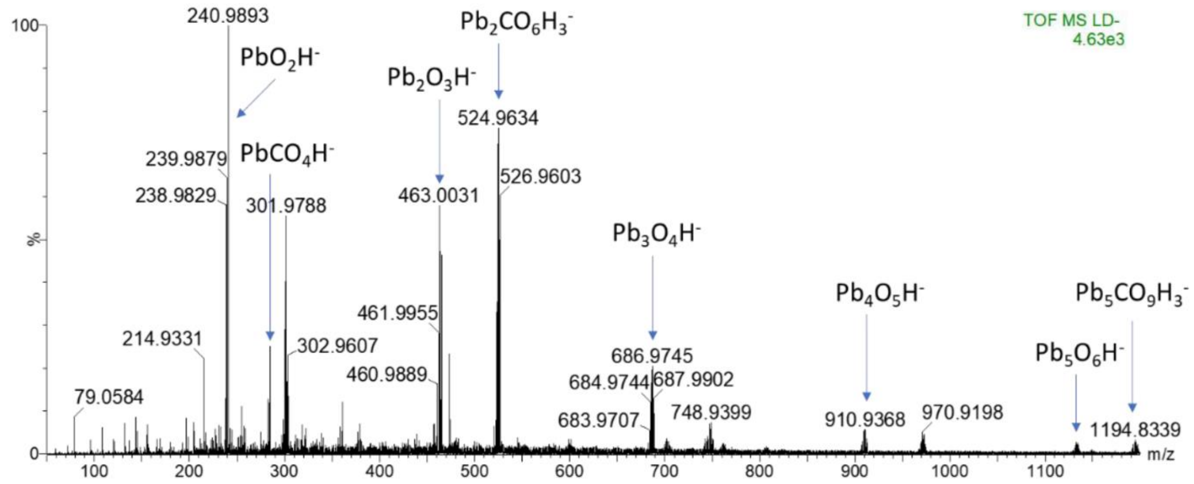

Figure S3: *LDI-MS spectra of the blue powder in negative ion mode (full range).*

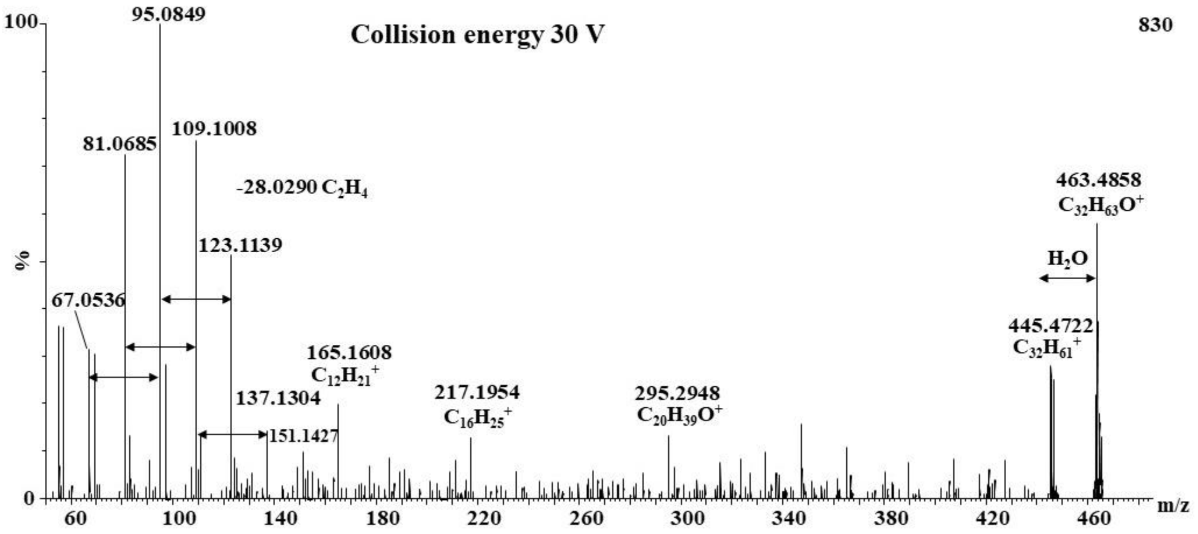

Figure S4*: MS/MS spectra of 463.4858 Da in pink powder.*

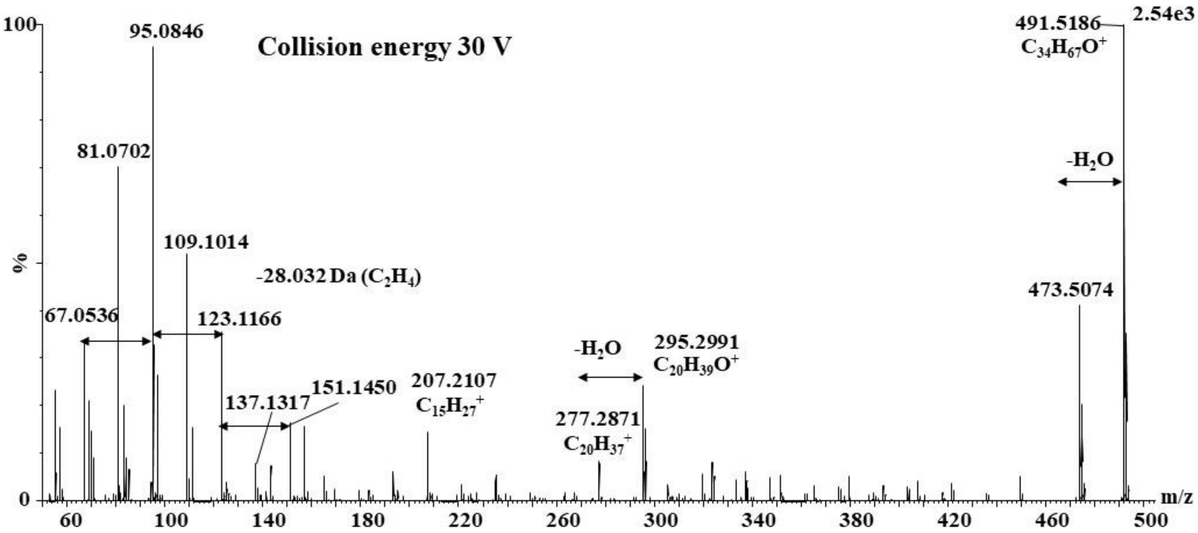

Figure S5: *MS/MS spectra of 491.5186 Da in pink powder.*

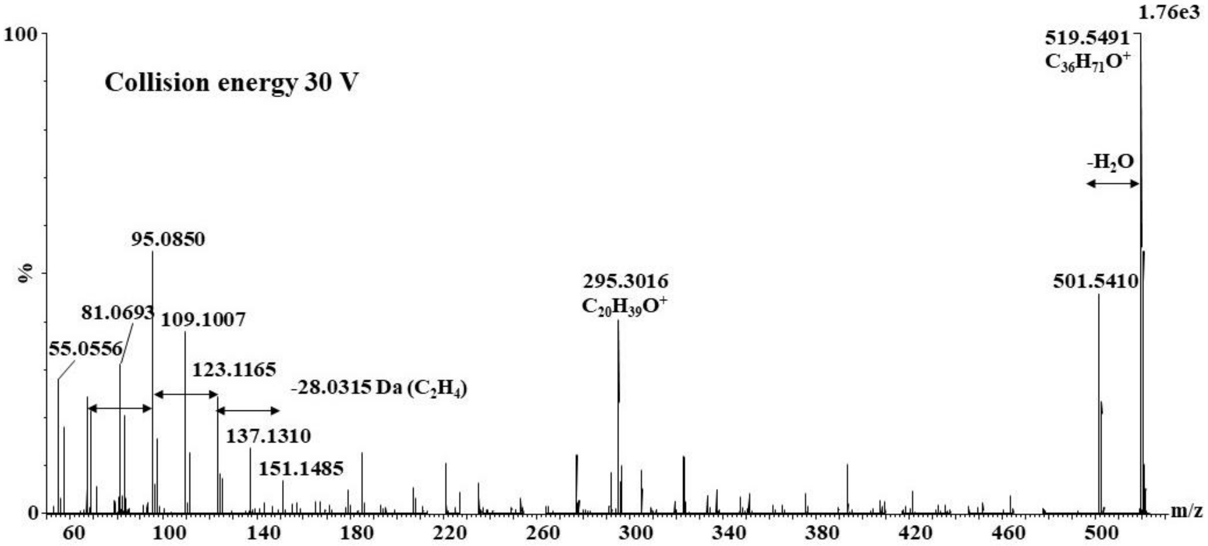

__Figure S6:__ MS/MS spectra of 519.5491 Da in pink powder.

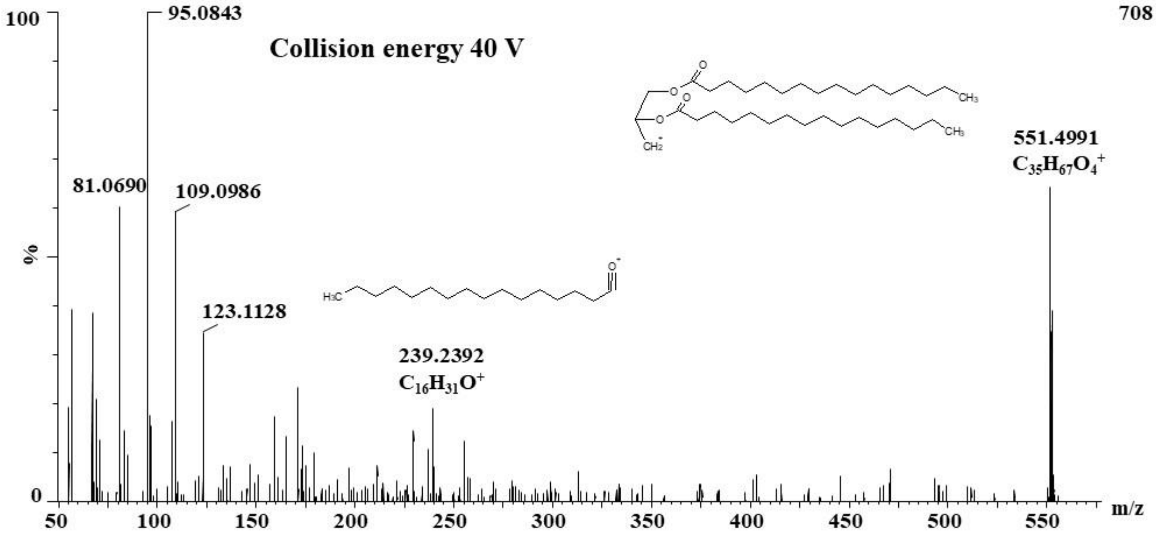

__Figure S7:__ MS/MS spectra of 551.4991 Da in pink powder.

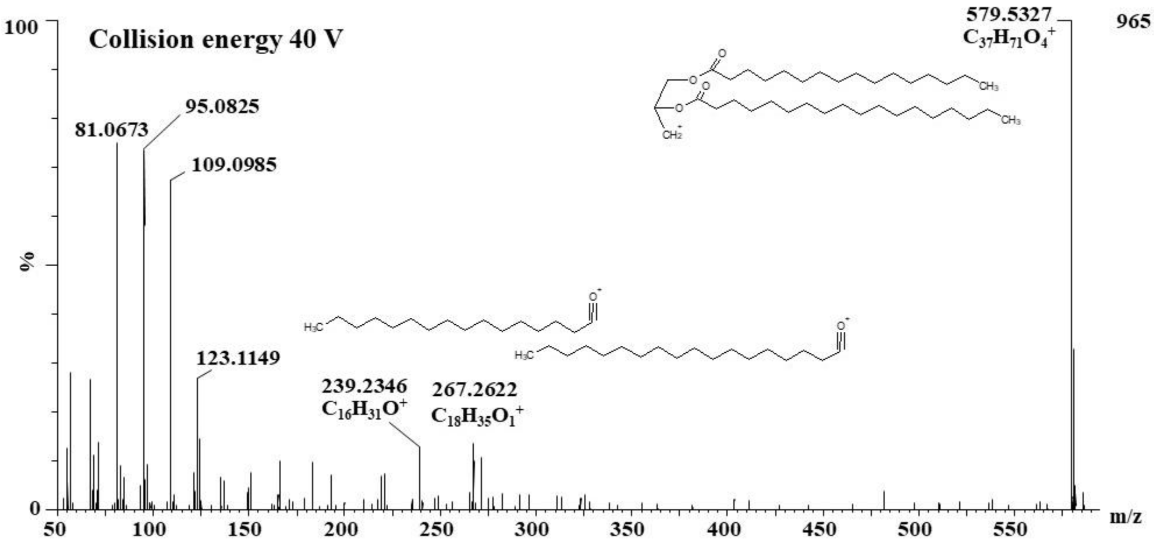

Figure S8: *MS/MS spectra of 579.5327 Da in pink powder*.

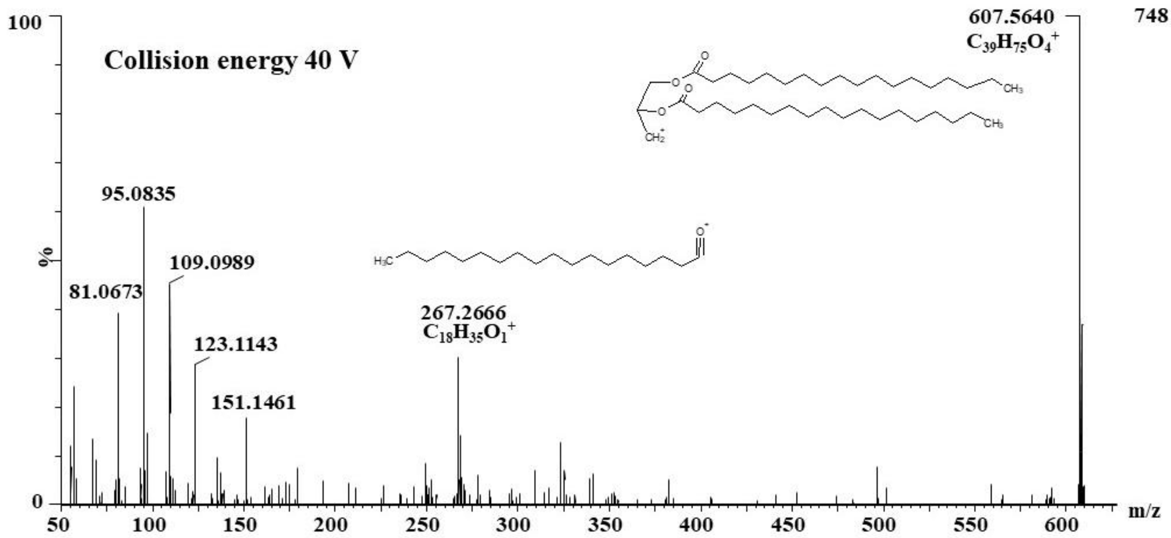

Figure S9: *MS/MS spectra of 607.5640 Da in pink powder*.

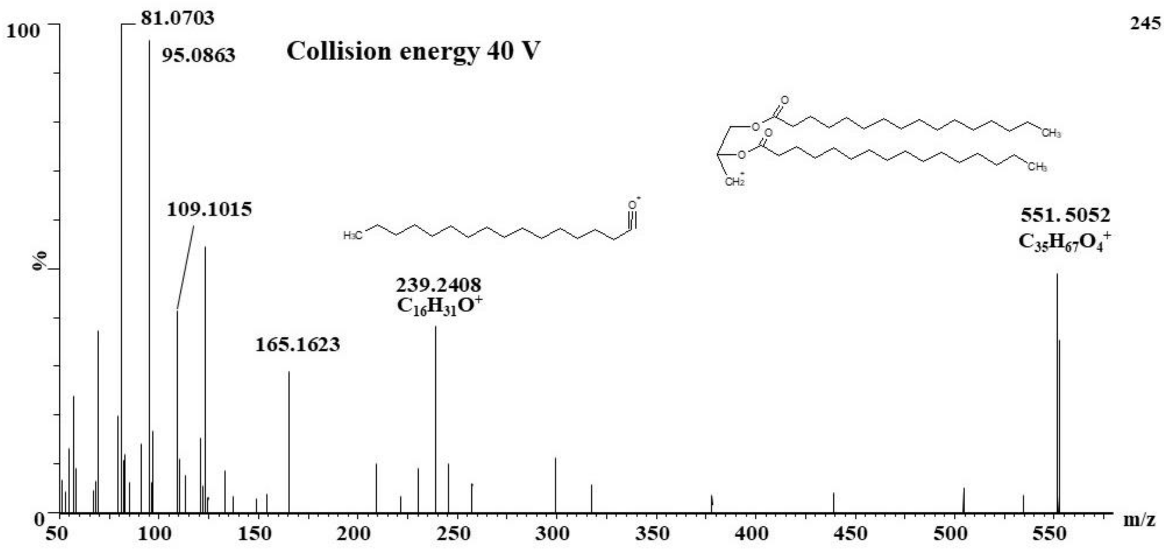

Figure S10: *MS/MS spectra of 551.4991 Da in blue powder.*

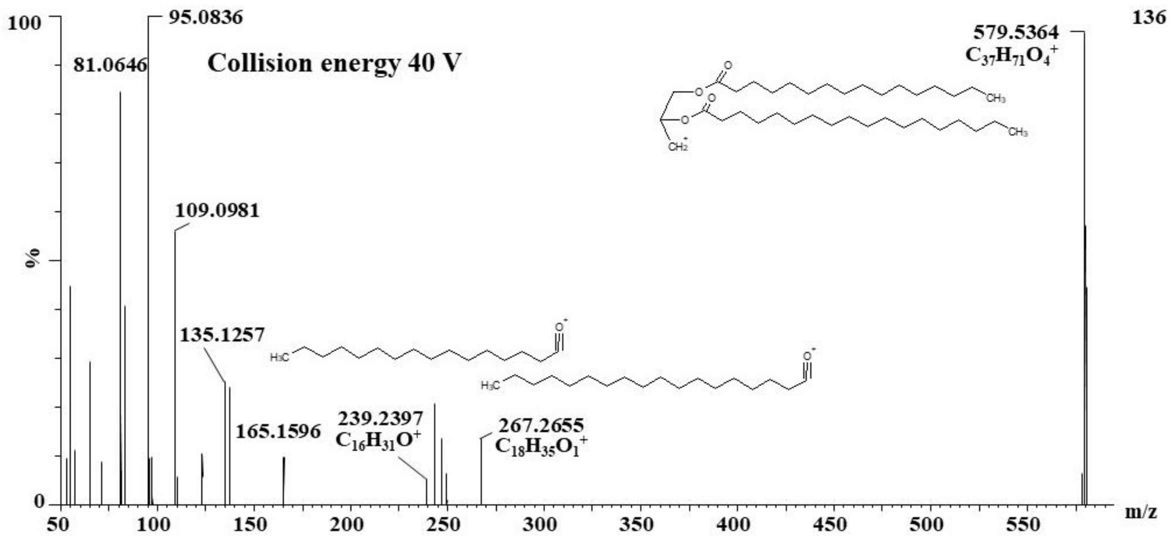

Figure S11: *MS/MS spectra of 579.5327 Da in blue powder.*

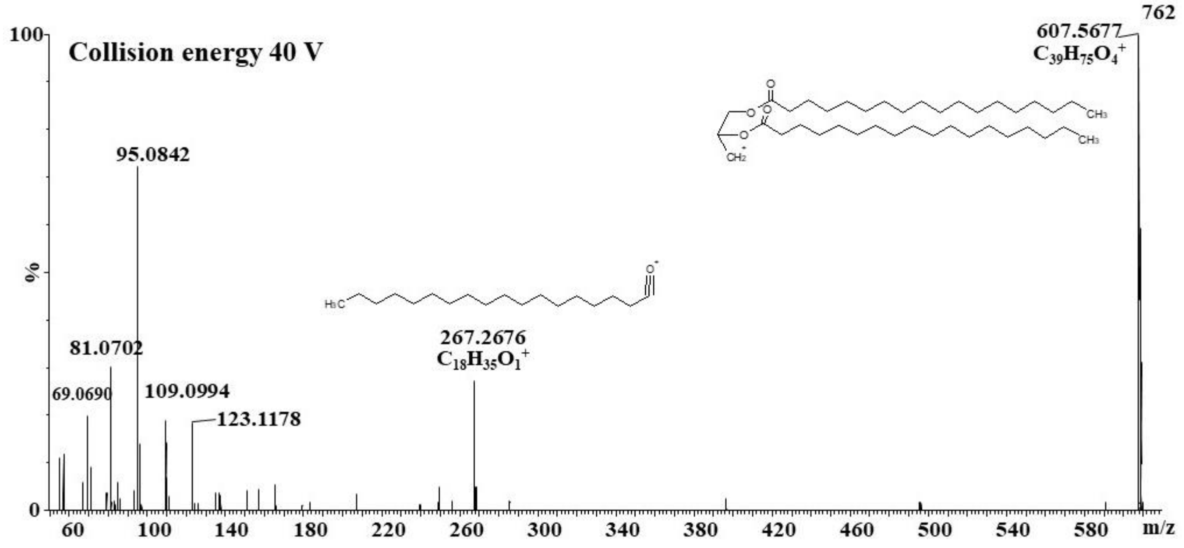

Figure S12: *MS/MS spectra of 607.5677 Da in blue powder.*

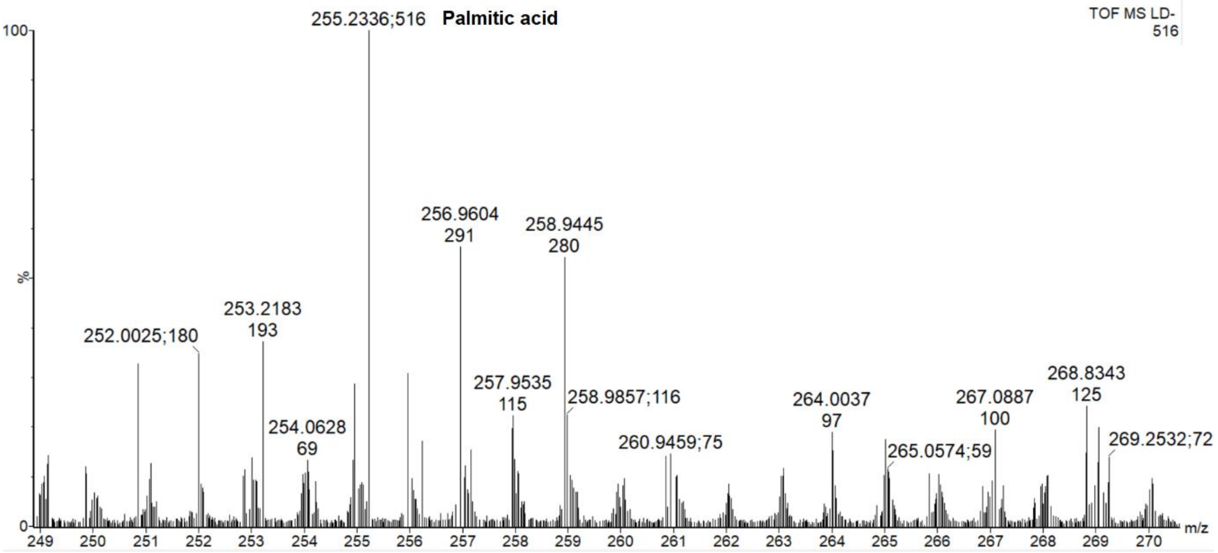

Figure S13: *LDI-MS spectra of the blue powder in negative ion mode (zoomed range 250–270 Da; lock-mass correction using PbO_2H^- clusters).*

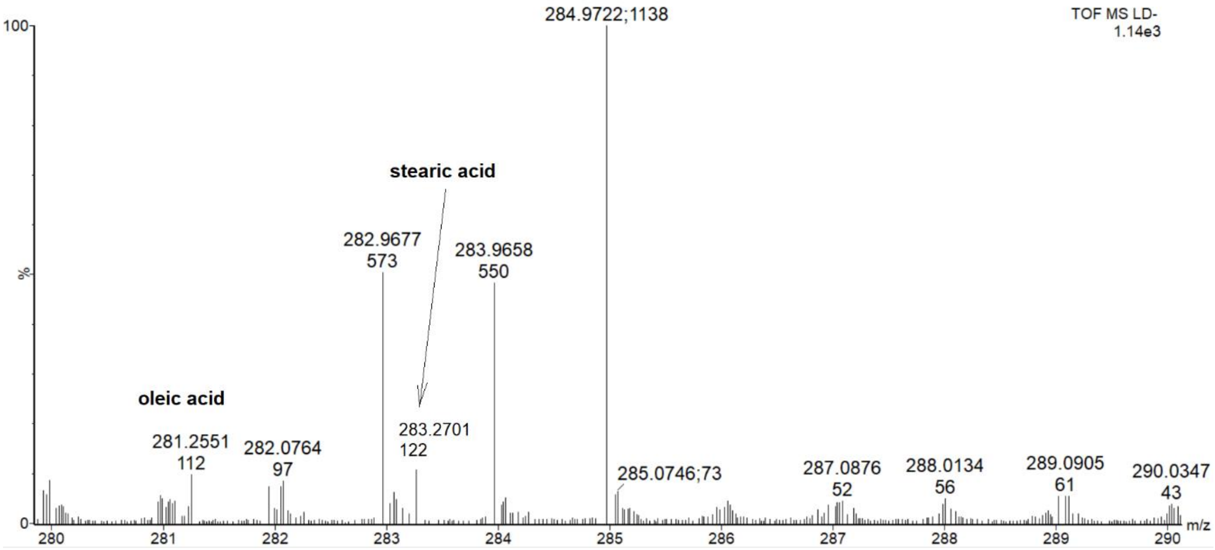

Figure S14: *LDI-MS spectra of the blue powder in negative ion mode (zoomed range 280–290 Da; lock-mass correction using* PbO_2H^- *clusters).*
